# Empirical research on folk moral objectivism

**DOI:** 10.1111/phc3.12589

**Published:** 2019-07-05

**Authors:** Thomas Pölzler, Jennifer Cole Wright

**Affiliations:** ^1^ University of Graz; ^2^ College of Charleston

## Abstract

Lay persons may have intuitions about morality's objectivity. What do these intuitions look like? And what are their causes and consequences? In recent years, an increasing number of scholars have begun to investigate these questions empirically. This article presents and assesses the resulting area of research as well as its potential philosophical implications. First, we introduce the methods of empirical research on folk moral objectivism. Second, we provide an overview of the findings that have so far been made. Third, we raise a number of methodological worries that cast doubt upon these findings. And fourth, we discuss ways in which lay persons' intuitions about moral objectivity may bear on philosophical claims.

## 1 INTRODUCTION

Look at the computer screen in front of you. Chances are that it is rectangular. Being rectangular is a paradigmatically objective property (see Huemer, 2005). What makes a thing have this property is that it has four sides and equal angles. This implies that the property's exemplification does not depend on what anybody thinks about it. Even if you yourself believed that the screen in front of you is round, or we all believed that it is round it would still actually be rectangular.^1^

What about morality? Is it objective too? Moral objectivists favor an affirmative answer. In their view, things are morally right, wrong, good, bad, etc. irrespectively of what anybody thinks about them (e.g., Brink, 1989; Huemer, 2005; Shafer‐Landau, 2003). Nonobjectivists, in contrast, deny moral objectivity. Some of them argue that moral properties do not exist at all (like the property of being the Loch Ness Monster; e.g., Blackburn, 2000; Joyce, 2001; Mackie, 2011). Others grant their existence but regard them as subjective. For example, it has been suggested that what makes an action right is that I myself believe that it is right (akin to matters of taste; e.g., Sartre, 1973) or that one's culture dominantly believes that it is right (akin to social conventions; e.g., Harman, 1996).

Proponents of the above debate have put forward numerous arguments for and against morality's objectivity. Some of these arguments appeal to lay persons' intuitions (e.g., Brink, 1989; Dancy, 1986; Enoch, 2017a, 2017b; Huemer, 2005; McNaughton, 1988). So far, philosophers' evidence for these empirical claims has been surprisingly weak. Sometimes, they have not provided any evidence at all. Moreover, when evidence has been cited, it has often been derived from introspection (e.g., Dancy, 1986; McNaughton, 1988) and/or unsystematic observations in classrooms or immediate social surroundings (e.g., Brink, 1989)—sources that, in our view, merit a fair amount of skepticism. This suggests that there is a need for rigorous scientific research on folk intuitions about moral objectivity.

For a long time, empirical scientists have only addressed folk moral objectivity indirectly or as a by‐product, such as in the context of moral/conventional tests (which involve an authority–independence question; e.g., Smetana, 1981; Turiel, 1983). In the last two decades, however, interest in this topic has steadily increased. More and more researchers have set out to measure explicitly what and how the folk think about morality's objectivity. This article introduces and assesses the resulting area of empirical research as well as its philosophical implications. First, we introduce the methods of empirical research on folk moral objectivity. Second, we provide an overview of the findings that have so far been made. Third, we raise a number of methodological worries that cast doubt upon these findings. And fourth, we discuss ways in which lay persons' intuitions about moral objectivity may bear on philosophical claims.

## 2 METHODS

The most challenging part of any study on folk moral objectivity is to come up with valid measures of this construct. So far, researchers have mainly used two kinds of measures: disagreement measures and truth‐aptness measures. In what follows, we will illustrate these measures by the example of one of the most influential studies on folk moral objectivity, which was conducted by Goodwin and Darley (2008). Then, we will briefly sketch some alternative measures.

### 2.1 Disagreement measures

Disagreement measures ask subjects for their interpretation of cases of moral disagreement. Each answer option is supposed to entail either (variants of) objectivism or nonobjectivism. Goodwin and Darley, for example, informed subjects that some other participant of their study had denied a moral statement that they themselves had agreed to; for example, the statement “Robbing a bank in order to pay for an expensive holiday is a morally bad action” (Goodwin & Darley, 2008, p. 1361) or “Scientific research on embryonic human stem cells that are the product of in vitro fertilization is morally permissible” (Goodwin & Darley, 2008, p. 1362). Then, subjects had to choose one of the following four answer options:

1. The other person is surely mistaken.
2. It is possible that neither you nor the other person is mistaken.
3. It could be that you are mistaken, and the other person is correct.
4. Other (Goodwin & Darley, 2008, p. 1344).

Goodwin and Darley (2008) interpreted “The other person is surely mistaken” responses as indicative of objectivism, and both “It is possible that neither you nor the other person is mistaken” and “It could be that you are mistaken, and the other person is correct” responses as indicative of subjectivism.

### 2.2 Truth‐aptness measures

Truth‐aptness measures investigate whether subjects consider (particular) moral statements to be truth‐apt, i.e., either true or false. In their 2008 study, Goodwin and Darley used two such measures. The first one asked subjects whether a moral statement was “true,” “false“ or “an opinion or attitude“:

How would you regard the previous statement? Circle the number. (1) True statement. (2) False statement. (3) An opinion or attitude. (Goodwin & Darley, 2008, p. 1344)

Goodwin and Darley's second truth‐aptness measure asked subjects whether there can be a correct answer as to whether a moral statement is true:

According to you, can there be a correct answer as to whether this statement is true? [yes] [no] (Goodwin & Darley, 2008, p. 1351)

As Goodwin and Darley (2008) interpreted their results, “true”/“false” and “yes” responses indicated objectivist intuitions, while “opinion and attitude” and “no” responses indicated subjectivist intuitions.

### 2.3 Alternative measures

In addition to disagreement and truth‐aptness measures, some researchers have recently experimented with novel ways of measuring intuitions about moral objectivity as well.

The most popular alternative approach proceeds by having subjects rate or choose among theoretical descriptions of (variants of) moral objectivism and nonobjectivism. For example, subjects were presented with statements such as “There exists a single moral code that is applicable to everyone, regardless of any individual person's beliefs or cultural identity” (Sarkissian & Phelan, 2019), “The only actions that are ultimately morally right or wrong are those actions that God prescribes” (Zijlstra, forthcoming‐b), or “When a person says that something is morally right or wrong, good or bad, etc. she intends to state a fact. Such facts exist—and they are independent from what anybody thinks about them” (Pölzler & Wright, forthcoming‐b).

Other alternative measures have been developed as well. Drawing on intuition‐pumps by Enoch, Zijlstra (forthcoming‐b) asked subjects whether a certain kind of joke with moral content is funny and whether a moral statement would still be true if our beliefs and practices had been very different. Wright and Pölzler (forthcoming) had their subjects choose among metaphors that correspond to variants of objectivism and nonobjectivism (such as that moral facts are “discovered,” “divine commandments,” “cultural inventions,” “individual inventions,” and “illusions”), and among comparisons of morality to nonmoral domains (science or mathematics, religion, social conventions, personal taste or preferences, superstition, and exclamations).

## 3 RESULTS

Based on the above measures, researchers have so far conducted several dozen studies on folk moral objectivity. In what follows, we will summarize the results of these studies. In particular, we will address the content, correlates, causes, and consequences of lay persons' metaethical intuitions.

### 3.1 Content

Some early studies on folk moral objectivity reported a strong tendency towards objectivism. Goodwin and Darley, for example, concluded that “[i]ndividuals seem to identify a strong objective component to their core ethical beliefs [...]. Arguably, many of our participants viewed their ethical beliefs as true in a mind‐independent way” (Goodwin & Darley, 2008, p. 1359; see also Nichols & Folds‐Bennett, 2003; Wainryb, Shaw, Langley, Cottam, & Renee, 2004).

Examining these studies' results more closely, however, makes it less clear whether this interpretation is appropriate (Pölzler, 2018b). Take again Goodwin and Darley's study. In this study, almost 30% of subjects' responses to the disagreement measure and almost 50% of their responses to the truth‐aptness measure fell on the option that the researchers took to be indicative of subjectivism (Goodwin & Darley, 2008, pp. 1347, 1351). Moreover, while some moral statements were dominantly classified as objective (e.g., the above statement about robbery), many others were dominantly classified as nonobjective (e.g., the stem cell research statement). This suggests that subjects in Goodwin and Darley's study may have actually favored what Wright, Grandjean, and McWhite (2013) called “metaethical pluralism,” i.e., they sometimes sided with objectivism and other times with nonobjectivism.

More recent studies have by and large confirmed this hypothesis of folk metaethical pluralism. Wright et al. (2013) and Wright, McWhite, and Grandjean (2014), for example, replicated Goodwin and Darley's results, using the exact same measures, but letting subjects classify the presented statements as moral and nonmoral themselves. Objectivity ratings for statements that were dominantly self‐classified as moral varied between as little as 5% and as much as 85%. Research based on different measures yielded high proportions of intrapersonal variation as well (e.g., Beebe, 2014; Beebe, Qiaoan, Wysocki, & Endara, 2015; Beebe & Sackris, 2016; Fisher, Knobe, Strickland, & Keil, 2017; Goodwin & Darley, 2012; Heiphetz & Young, 2017; Wright, 2018; Zijlstra, forthcoming‐a).^2^

Folk metaethical pluralism can lean towards objectivism or nonobjectivism. In contrast to earlier research, several recent studies suggest that lay persons' pluralism exhibits a tendency towards nonobjectivism. Sarkissian, Park, Tien, Wright, and Knobe (2011), for example, presented subjects with cases of moral disagreement in which the disagreeing parties are members of different cultures. This significantly increased their nonobjectivist (relativist) responses. Pölzler and Wright (forthcoming‐b) pitted realism against even more forms of nonobjectivism, including also individual subjectivism, divine command theory,^3^ error theory, and non‐cognitivism. No less than 87% of subjects' responses to their abstract and 92% of responses to their concrete tasks were in favor of nonobjectivism.

### 3.2 Correlates

In addition to the content of lay persons' intuitions about moral objectivity, researchers have also claimed to have identified a number of variables that correlate with these intuitions. Table 1 provides an overview of these variables (for a similar overview see Colebrook, forthcoming).

**Table 1:** Variables that have been found to correlate with intuitions about moral objectivity in empirical research

Category	Variable	Direction
Cognition^a^	*Moral Judgement Strength*	The more strongly people agree with a moral statement, the more objectivist their intuitions about it (Beebe, 2014; Beebe, Qiaoan, Wysocki, & Endara, 2015; Goodwin & Darley, 2010, 2012; but see Goodwin & Darley, 2010; Wright, Grandjean, & McWhite, 2013).
	*Perceptions of Consensus*	The more widely subjects believe a moral statement to be accepted by other members of their society, the more objectivist their intuitions about it (e.g., Beebe, 2014; Beebe, Qiaoan, Wysocki, & Endara, 2015; Goodwin & Darley, 2008, 2012; Wright, McWhite, & Grandjean, 2014).
	*Religious Belief*	Justifying morality by reference to God (Goodwin & Darley, 2008, 2010, 2012, but see Wright, Grandjean, & McWhite, 2013), belief in a punishing (as opposed to a loving) God (Sarkissian & Phelan, 2019), divinity concepts (Yilmaz & Bahçekapili, 2015) and strength of religious belief (Pölzler & Wright, forthcoming‐b) correlate with objectivism.
	*Disgust*	The more people feel disgusted by other cultures' practices, the more objectivist their intuitions about these practices (Cameron, Payne, & Doris, 2013).
	*Mortality Salience*	The more people are aware of their mortality, the more objectivist their intuitions (Yilmaz & Bahçekapili, 2018).
	*Desire to Punish*	The stronger people's desire to punish a moral transgressor the more objectivist their intuitions about his/her action (Rose & Nichols, forthcoming).
	*Argumentative Mindset*	The more people argue to win (as opposed to argue to learn) the more objectivist their intuitions (Fisher, Knobe, Strickland, & Keil, 2017).
Behavior	*Tolerance/Comfort*	The lower people's tolerance for disagreeing others (Wright, Grandjean, & McWhite, 2013; Wright, McWhite, & Grandjean, 2014) and comfortableness about them (Goodwin & Darley, 2012; Wright, McWhite, & Grandjean, 2014) the more objectivist their intuitions.
	*Ethical Behavior*	The less likely people engage in unethical behavior (e.g., cheating, Rai & Holyoak, 2013) and refrain from engaging in ethical behavior (e.g., lottery, Young & Durwin, 2013) the more objectivist their intuitions.
Development	*Age*	Children have more objectivist intuitions than adults (Heiphetz & Young, 2017; Wainryb, Shaw, Langley, Cottam, & Renee, 2004). With adults, the degree to which they attribute objectivity varies across their lifespan (with the research being conflicting about what these changes look like; see, e.g., Beebe & Sackris, 2016 vs. Pölzler & Wright, forthcoming‐b).
Personality	*Openness to Experience*	The less open people are to experiences the more objectivist their intuitions (Feltz & Cokely, 2008; Goodwin & Darley, 2010).
	*Disjunctive Reasoning Ability*	The worse people's ability to consider alternative possibilities when deciding between options the more objectivist their intuitions (Goodwin & Darley, 2010).
Content	*Harmfulness*	Moral statements about harmful transgressions correlate with more objectivist intuitions than moral statements about non‐harmful or only symbolically harmful transgressions (Feltz & Cokely, 2008; Goodwin & Darley, 2010).
	*Judgment Valence*	Negative moral statements (e.g., wrong) correlate with more objectivist intuitions than positive moral statements (e.g., right; Beebe, 2014; Goodwin & Darley, 2010).

a We use the term “cognition” in a broad sense that also encompasses affective mental states and processes.

In what follows, we will explain to what extent the above variables have been found to *cause* intuitions about moral objectivity or to *be caused* by them.

### 3.3 Causes

Several variables' causal influence has been established experimentally. Most importantly, it has been found that increasing subjects' perceptions of consensus (Ayars & Nichols, 2019; Goodwin & Darley, 2012), priming them with divinity concepts (Sarkissian & Phelan, 2019; Yilmaz & Bahçekapili, 2015), inducing disgust (Cameron, Payne, & Doris, 2013), making their mortality more salient to them (Yilmaz & Bahçekapili, 2018), and a stronger desire to punish transgressors (Rose & Nichols, forthcoming) render their metaethical intuitions more objectivist.

All of these explanations of folk moral objectivism and nonobjectivism specify how people's intuitions come about within their lifetimes. Researchers who have addressed these intuitions' *ultimate* causes have typically argued that lay persons are moral objectivists (contradicting the results reported in Section 2.1) and that this objectivism is a biological adaption. According to Ruse (1998) and Joyce (2006), for example, interpreting moral obligations as objective increased our ancestors' reproductive success by making them more likely to meet these obligations, and hence to cooperate with others (for a more complex evolutionary account see Stanford, 2018).

### 3.4 Consequences

Some of the variables identified in Section 2.2 have also been claimed to be consequences of people's intuitions about moral objectivity. Subjects who were primed with objectivism were found to behave more ethically than subjects who were primed with subjectivism, for example, in that they were less likely to cheat in a lottery and to state that they would be willing to steal (Rai & Holyoak, 2013), and more likely to donate to charity (Young & Durwin, 2013). Increased objectivism also led to stronger belief in God (Yilmaz & Bahçekapili, 2015) and to thinking of God as punishing (Sarkissian & Phelan, 2019). Finally, suggesting a bidirectional causal relationship, Wright et al. (2013) and Wright et al. (2014), and Pölzler and Wright (forthcoming‐c) argue that just as being less tolerant and comfortable with divergent beliefs and practices regarding moral issues leads people to adopt objectivist interpretations of these issues, objectivist interpretations lead people to be less tolerant, and less comfortable with divergent beliefs and practices as well.

## 4 WORRIES

So far, we have discussed the extant empirical research on folk moral objectivism as if it were (largely) reliable and valid. On closer consideration, however, this assumption turns out to be doubtful. While this area of research does not fare too badly in terms of reliability and external validity, it has rightly become subject to another—more fundamental—worry. Many studies on folk moral objectivism may lack in internal validity; i.e., they may have failed to measure what they purported to measure, namely intuitions about *moral objectivism*.

### 4.1 Reliability

Psychology has recently been shaken by a methodological crisis. In a large‐scale collaborative attempt, only 36.1% of its findings could be replicated (Open Science Collaboration, 2015). In contrast and in line with experimental philosophy research in general (Cova, Strickland, et al., 2018),^4^ the replication rate of studies on folk moral objectivism appears reasonably high.

As argued in Section 2.1, Goodwin and Darley's influential 2008 study suggests that lay persons tend towards metaethical pluralism. This finding about the content of lay persons' metaethical intuitions has been replicated several times, making it possible to be confident that it is not simply an artifact of the data or the participants involved. For example, utilizing the same disagreement and truth‐aptness measures as Goodwin and Darley, Wright, Grandjean, and McWhite (2013), Wright, McWhite, and Grandjean (2014), and Wright (2018) found similarly high degrees of variation in subjects' objectivist versus nonobjectivist interpretation of moral statements. Sarkissian, Park, Tien, Wright, and Knobe's (2011) disagreement‐based study has recently been successfully replicated in children (Schmidt, Gonzalez‐Cabrera, & Tomasello, 2017) and adults (Gonzalez‐Cabrera, personal communication, manuscript in progress) as well.

Some findings about metaethical intuitions' correlates, causes, and consequences have been stably replicated as well. This first and foremost holds for the connection between moral objectivism and higher levels of attitudinal and behavioral intolerance (Wright, 2018; Wright, Grandjean, & McWhite, 2013; Wright, McWhite, & Grandjean, 2014). In contrast, the replicability of studies which purported to manipulate metaethical intuitions (e.g., using religious, mortality, and disgust primes (Cameron, Payne, & Doris, 2013; Sarkissian & Phelan, 2019; Yilmaz & Bahçekapili, 2015, 2018)) is less clear.^5^

### 4.2 External validity

Another potential worry about research on folk moral objectivism concerns its generalizability. Some studies' samples have mostly involved US students (who tend to be more educated, more liberal, younger, etc. than most people; e.g., Goodwin & Darley, 2008; Nichols, 2004). Researchers have sometimes used unduly unrealistic or humorous experimental stimuli (involving, for example, actions like killing one's child because it is ugly or characters such as pentagon‐obsessed aliens; e.g., Sarkissian, Park, Tien, Wright, & Knobe, 2011). Finally, subjects were also often only asked to rate item statements of a particular kind: statements that involve thin moral concepts (rather than also thick concepts, such as just, courageous and cruel), that are about actions (rather than persons or states of affairs), and that concern kinds of cases (rather than particular cases or principles; e.g., Beebe & Sackris, 2016; Goodwin & Darley, 2008; Nichols, 2004).

The above considerations suggest that the external validity of studies on folk moral realism can and should indeed be improved. However, it is important to note that it does not seem to be extremely low either. There are studies involving diverse age groups (e.g., Beebe & Sackris, 2016; Nichols & Folds‐Bennett, 2003; Wainryb, Shaw, Langley, Cottam, & Renee, 2004) and nonstudent subjects from Amazon Mechanical Turk (e.g., Wright, 2018; Zijlstra, forthcoming‐a, forthcoming‐b). There is cross‐cultural research (Beebe, Qiaoan, Wysocki, & Endara, 2015; Sarkissian, Park, Tien, Wright, & Knobe, 2011). And there are studies that use more heterogeneous and realistic item statements (e.g., Goodwin & Darley, 2012; Pölzler & Wright, forthcoming‐b). Our main worry about research on folk moral objectivism rather concerns its internal validity.

### 4.3 Internal validity

Studies in this area purport to measure whether lay persons are objectivists in the philosophical sense of endorsing mind‐independent moral properties (e.g., Goodwin & Darley, 2008; Nichols, 2004). There at least five reasons to doubt that this is what the studies have actually measured.

First, most studies have failed to account for or appropriately disentangle main metaethical positions. This may have distorted their results. Recall, for example, Goodwin and Darley's disagreement measure (Section 2.1). This measure's first answer option (“The other person is surely mistaken”) is actually not only entailed by objectivism but also by several versions of subjectivism. Cultural relativists, for example, hold that an action is right if and only if it is dominantly deemed right within one's culture. The moral disagreements that Goodwin and Darley's subjects were presented with took place within a particular culture. But within one particular culture there can only be one dominant view about an action's rightness. Thus, even cultural relativists should have held that in these cases one disagreeing party (presumably the “other person”) is mistaken (Pölzler, 2018b; Sarkissian, Park, Tien, Wright, & Knobe, 2011).

Second, researchers have sometimes conflated moral objectivism and nonobjectivism with distinct metaethical positions. Take Goodwin and Darley's truth‐aptness measures. These measures purported to illuminate intuitions about moral objectivity. However, while denials of (correspondence‐theoretic) truth‐aptness indeed entail non‐objectivism, affirming truth‐aptness does not necessarily indicate objectivism. Some variants of nonobjectivism are consistent with considering a moral statement to be “true” or ”false,” or with thinking that “there can be a correct answer as to whether this statement is true” as well. In particular, subjectivists believe that moral sentences are true or false depending on whether they correctly represent the subjective moral facts, and error theorists believe that all moral sentences are false. By pitting truth‐aptness against non‐truth‐aptness Goodwin and Darley therefore did not measure the proportion of objectivists versus nonobjectivists among their subjects but at best the proportion of cognitivists versus non‐cognitivists (Pölzler, 2017, 2018a, 2018b).

Third, some studies may have failed to (fully or exclusively) measure moral objectivism because responses in these studies can also be (partly) explained by irrelevant nonmetaethical intuitions. Goodwin and Darley's first truth‐aptness task, for example, required subjects to think about whether they take statements such as “Scientific research on embryonic human stem cells that are the product of in vitro fertilization is morally permissible” to be *either* true *or* false (instead of merely truth‐*apt*). Their answer may thus have been based on first‐order moral intuitions. Those who opted for “true” and “false” may not have done so because they regarded the statement as truth‐apt, but rather because they think that stem cell research is or is not morally permissible (Beebe, 2015; Pölzler, 2018a, 2018b).

Fourth, research on folk moral objectivism assumes an understanding of moral truth, rightness, correctness, etc. that may not correspond to the folk's ordinary understanding of these notions. In order for disagreement and truth‐aptness measures to indicate intuitions about objectivism the notions of moral truth, rightness, correctness, etc. must be interpreted in a correspondence‐theoretic sense, i.e., subjects must hold that to say of a disagreeing party that he or she is right about a moral statement or of such a statement that it is true is for these statements to correctly represent moral facts (rather than, say, to merely reaffirm these statements). But there is no evidence that subjects in studies on folk moral objectivism have indeed share this understanding (Pölzler, 2018a, 2018b).

Fifth and finally, contrary to what has been argued above, suppose that studies' answer choices did fully and exclusively logically entail (variants of) moral objectivism and nonobjectivism. Even then, their internal validity may not be high. This is because subjects who choose a particular answer option in such studies need not have an intuition in favor of the entailed variant of objectivism or nonobjectivism at all. Their responses can be explained in at least three plausible alternative ways as well. First, subjects may lack an intuition about the particular question that they are asked—such as about how to interpret a case of disagreement or about whether a moral statement is “true,” “false” or “an opinion or attitude”—and simply supply whatever response seems most reasonable (see Bengson, 2013). Second, even if they have such an intuition, their response may not accurately reflect it (say, because they interpret materials in unintended ways or are inattentive or confused; see Bengson, 2013; Moss, 2017). And third, even if subjects both have an intuition about a study's moral disagreement or truth‐aptness question and their answer accurately reflects this intuition, they may still lack any (determinate, robust, etc.) intuition about moral objectivity as such. This underlying metaethical matter may simply be too abstract or complex for any view about it to seem true to lay persons (Sinclair, 2012).

In recent years, several researchers have begun to address some of the five worries explained above. For example, they have accounted for cultural relativism (e.g., Pölzler & Wright, forthcoming‐b; Sarkissian, Park, Tien, Wright, & Knobe, 2011); excluded or more properly interpreted truth‐aptness measures (e.g., Pölzler & Wright, forthcoming‐b); used alternative measures (e.g., Pölzler & Wright, forthcoming‐b; Sarkissian, Park, Tien, Wright, & Knobe, 2011; Zijlstra, forthcoming‐a, forthcoming‐b); provided subjects with instructions about metaethics or relevant metaethical debates (Pölzler & Wright, forthcoming‐b; Wright, 2018); included (more) attention checks, comprehension checks and/or response time measures (e.g., Pölzler & Wright, forthcoming‐b; Sarkissian & Phelan, 2019); and asked subjects to verbally explain their responses (e.g., Goodwin & Darley, 2008; Wright, Grandjean, & McWhite, 2013; Wright, McWhite, & Grandjean, 2014).

Any such attempt is to be welcomed. However, the number of these more promising studies is still rather small, and even they are vulnerable to a number of criticisms.^6^ We therefore believe that before conclusions such as those presented in Section 3 can be drawn with confidence more research needs to be conducted.

## 5 SIGNIFICANCE

Knowing about the content, causes, and consequences of lay persons' intuitions about moral objectivity is obviously desirable from a psychological perspective. But is the above research also relevant to philosophy? And if yes, in what ways? In this Section, we will present and evaluate four arguments that attempt to draw philosophical conclusions from hypotheses about folk moral objectivism: the debunking argument, the presumptive argument, the conceptual argument, and the methodological argument. Note that many of these arguments and their variants need not be taken to be about intuitions in a narrow philosophical sense of the term, but may be reformulated in terms of beliefs. We will hence mostly skip discussions about the existence and general nature of intuitions (for such discussions see, e.g., Machery, 2017; Pust, 2000).

### 5.1 The debunking argument

Most of the factors that have so far been claimed to cause lay persons' intuitions about moral objectivity (see Section 3.3) appear to be independent of the truth about this matter. Take, for example, one's desire to punish. A person who strongly desires to punish would do so even if objectivism were false, and a person who does not desire to punish at all would do so even if objectivism were true. This means that if the strength of our desire for punishment leads us to hold a true belief about moral objectivism, we are just lucky to have arrived at this true belief. Given this element of epistemic luck, it might be argued that our beliefs about moral objectivism lack justification—that the above empirical research “debunks” them (in the same way in which some philosophers have attempted to debunk some or all first‐order moral beliefs; e.g., Greene, 2008; Joyce, 2006).

Debunking arguments can target all metaethical beliefs or only beliefs in (certain variants of) objectivism or nonobjectivism. The above argument—that has been put forward by Rose and Nichols (forthcoming)^7^—is global. If it were sound then people would not be justified in believing either objectivism or nonobjectivism. A local version can be found in Ruse (1998). According to him, intuitions in favor of objectivism would have led our ancestors to reproduce more successfully even if objectivism were false. We are thus unjustified to believe in objective moral truths. However, Ruse argues that there are still good reasons for accepting nonobjectivism.

Proponents of debunking arguments face several challenges. For example, they need to establish that the identified causes of our intuitions about objectivism are really independent of objectivism's truth,^8^ and that these causes are dominant (so that other causes cannot bring the intuitions back on track). Critics may also doubt that these arguments apply to philosophers. They may point out that, in contrast to lay persons, philosophers' intuitions about objectivism are not subject to (strong) irrelevant influences. Philosophers do not even seem to primarily ground their beliefs about moral objectivity in intuitions about this matter in the first place. They also and maybe dominantly engage in more theoretical considerations, such as about ontological parsimony, coherence with broader philosophical claims, etc.

In response to this last objection proponents of debunking arguments may claim that metaethicists' theoretical considerations are (primarily) *post hoc* rationalizations of their prior intuitions. Moreover, philosophers' intuitions about several subject matters have been found to be susceptible to irrelevant influences (e.g., Schulz, Cokely, & Feltz, 2011; Schwitzgebel & Cushman, 2012; Tobia, Buckwalter, & Stich, 2013; Löhr, 2019; for an overview see Machery, 2017), so it is certainly possible that they would likewise be distorted when it comes to moral objectivity.

### 5.2 The presumptive argument

How things seem to us is not always how they in fact are. Yet most metaethicists agree that the intuitiveness of a position at least grounds a presumption in its favor. Arguments of this kind have most often been put forward by objectivists. They claim that as objectivism seems true to us, we have a *prima facie* reason to believe in it. Objectivism should be our “metaethical starting point” (Brink, 1989, p. 24).

Why would the intuitiveness of objectivism or nonobjectivism ground a presumption in its favor? This question has been answered in several different ways (see Loeb, 2008; Pölzler, forthcoming). For example, it has been argued that we have a *prima facie* reason to believe in objectivism because it best explains our intuitive tendency towards objectivism (explanatory version; e.g., Brink, 1989); because it best justifies or vindicates this tendency (justificatory version; Shafer‐Landau, 2003)^9^; or because we have a *prima facie* reason to believe *every* proposition that seems true to us (phenomenal conservatist version; e.g., McNaughton, 1988).

These arguments' underlying empirical hypotheses differ. For example, while proponents of the explanatory version often appeal to intuitions that are implicit (such that we are unaware of and unable to verbalize them) or reflective (what things seem like after some reflection; e.g., Brink, 1989), the phenomenal conservatist argument is more readily associated with immediate intuitions (what things seem like at first glance; e.g., McNaughton, 1988). For different versions of the presumptive argument to succeed empirically, they hence require different kinds of evidence, provided by different kinds of studies on folk moral objectivism.

Philosophically, all versions of the presumptive argument appear to be on shaky grounds (Loeb, 2007; Pölzler, forthcoming). Take, for example, the justificatory version. It is not obvious that being able to hold on to our metaethical intuitions is desirable. Whatever these intuitions look like, they may well do us more harm than good. Even more importantly, why should objectivism or nonobjectivism be measured in terms of their practical consequences at all? Metaethics rather seems to be a theoretical endeavor aiming to discover philosophical truths (Pölzler, forthcoming).

### 5.3 The conceptual argument

Most metaethicists assume that claims about the meaning of moral concepts are justified to the extent to which they conform to lay persons' conceptual intuitions (often understood as their pretheoretical dispositions to apply or refrain from applying these concepts; e.g., Jackson, 1998; Kauppinen, 2007; Loeb, 2008). If this assumption is correct, then research on folk moral objectivism may be relevant to conceptual analysis. After all, truth‐aptness tasks directly measure conceptual intuitions^10^; and intuitions about moral objectivism as a whole imply such intuitions (e.g., affirming the existence of objective moral facts implies affirming that our moral judgements purport to refer to these facts).

The main objection against empirical arguments of this kind concerns the targeted kind of intuitions. In order for intuitions to be relevant to the analysis of moral concepts, critics have argued, they need to be reflective (as opposed to immediate), semantic (as opposed to pragmatic), and representing conceptual competency (originating from speakers who do not apply moral concepts in obviously wrong ways).^11^ For methodological and even logical reasons (quantitative) empirical studies have been claimed to be inapt to uncover intuitions of this kind (see Kauppinen, 2007; Ludwig, 2007, 2010; Sosa, 2009).

The above objection's strength significantly depends on one's interpretation of the reflectiveness, semantics, and competency requirements (Hannon, 2018). Their strongest (logical) versions most likely fail (e.g., Nadelhoffer & Nahmias, 2007). Moreover, in our view, methodological devices such as instructions (that promote reflection), comprehension checks (that test reflection and competency), and open‐ended questions (that test reflection, semantic content, and competency) enable researchers to measure or at least approximate relevant intuitions (see also, e.g., Horvath, 2010; Nadelhoffer & Nahmias, 2007; Sytsma & Livengood, 2015). Pölzler and Wright (forthcoming‐a), for example, have argued that the finding that most lay persons regard most moral sentences as truth‐apt under these more stringent conditions supports the conceptual claim that moral sentences actually are truth‐apt.

### 5.4 The methodological argument

In grounding their theories about moral objectivity, metaethicists sometimes appeal to their own intuitions (which they take to be representative). They use these intuitions as a methodological tool to help build these theories. For example, Brink (1989: 26) at one point claims that the form and content of our moral judgements supports objectivism because “[w]e do not say that murder is wrong for Spike.”

Research on folk moral objectivism suggests an important methodological requirement with regard to such arguments (Hopster, forthcoming; Sarkissian, 2017). According to much existing research, lay persons' intuitions vary with moral statements' content (such as with harmfulness and statement valence; Section 3.1). In appealing to armchair intuitions, objectivists and nonobjectivists therefore must not nourish their thinking with a “one‐sided diet” of examples (Wittgenstein, 2001: § 593). They need to consider several kinds of cases. For example, they need to think about whether people would also refuse to say that *euthanasia* is wrong for Spike; or about whether moral sentences about *smoking in public places* admit of such a subjectivist interpretation.

So far both objectivists and non‐objectivists have assumed that all moral sentences have the same meaning (Gill, 2009; Sinnott‐Armstrong, 2009). Our suspicion is that if one combines the above methodological requirement with the conceptual argument explained in the previous section then future empirical research will make this assumption look increasingly doubtful. We may find that while the intended truth‐makers of some moral sentences (such as about murder) are objective moral facts, other moral sentences (such as about smoking in public places) have subjective moral facts as their intended truth‐makers or are not truth‐apt at all. Such a “variantist” moral semantics could ground nontraditional metaethical positions, according to which different metaethical views are true for different parts of moral language and thought (thus, potentially vindicating the folk's purported metaethical pluralism).

## 6 CONCLUSION

Lay persons may have intuitions about morality's objectivity. On the face of it, empirical research suggests that these intuitions favor nonobjectivism‐leaning metaethical pluralism, and that they influence and are influenced by numerous cognitive, behavioral, personality‐related, developmental, and content‐related variables. But these findings must, at least for now, be treated with caution. Considering their methods, as it stands, it is unclear whether existing studies have actually succeeded in (fully and exclusively) measuring intuitions about moral objectivity, at least in a standard philosophical interpretation of “moral objectivity.” Future research in this area hopefully continues its recent trend of methodological maturation. Whatever its outcomes, this research will not only be psychologically interesting but may also have important implications for philosophy.

## ENDNOTES

1 For a more detailed account of moral objectivity *qua* mind‐independence see, e.g., Huemer, 2005; Joyce, 2007.

2 There is some evidence to suggest that lay persons' metaethical pluralism may even be reflected in their brain activity. Theriault, Waytz, Heiphetz, and Young (forthcoming) found that moral judgements varied in the extent to which they activated Theory of Mind Networks (ToMNs). Those judgements that had previously been rated as more fact‐like (i.e., objectivist) triggered ToMN processing less than those that had been rated as more preference‐like (i.e., subjectivist). This finding was robust even after controlling for a variety of semantic/syntactic features, as well as activation of mental states and mental imagery, whether a person was present, etc. While there are a number of potential explanations for the finding, one promising explanation offered by the authors themselves is that the objective, fact‐like moral claims may simply provide more predictable social information—thereby requiring less ToMN processing—than subjective, preference‐like morals, or preferences.

3 Divine command theory is more commonly classified as a variant of objectivism. For defenses of Pölzler and Wright's classification see, e.g., their above‐cited paper Pölzler and Wright (forthcoming‐b) as well as Huemer (2005). Moreover, note that even if divine command theory were regarded as objectivist most responses in Pölzler and Wright's study would still be in favor of nonobjectivism, namely, 77% in the abstract and 88% in the concrete condition. (In other words, only few subjects in this study were drawn to divine command theory.)

4 The XPhi‐replicability project's recent attempt to replicate 40 experimental philosophy studies resulted in a replication rate of about 70% (Cova, Strickland, et al., 2018).

5 Outside the domain of metaethical intuitions at least the effects of mortality salience are quite well established and consistent with the studies cited above (but see Wissink et al., forthcoming). In contrast, some studies about disgust's influence on moral judgements have failed to replicate. At the very least these studies seem to have yielded exaggerated effect sizes (see, e.g., Johnson, Cheung, & Donnellan, 2014; Landy & Goodwin, 2015).

6 For example, Pölzler and Wright's (forthcoming‐b) experimental design may have inflated the proportion of non‐objectivist responses by involving considerably more nonobjectivist than objectivist answer options.

7 Rose and Nichols take their argument to target moral universalism (rather than objectivism), i.e., the claim that true moral sentences are true for any individual at any time and any place. However, to measure moral universalism they use one of the same disagreement task that has been used to measure moral objectivism (from Sarkissian, Park, Tien, Wright, & Knobe, 2011).

8 For example, at first sight it might seem that beliefs about how widely people agree about a moral statement do not track the metaethical status of this statement. But Ayars and Nichols (2019) have recently argued that they do.

9 Note that Shafer‐Landau's argument is in favor of moral cognitivism rather than objectivism. However, analoguous objectivist versions of this argument have been discussed as well (e.g., Sinclair, 2012).

10 It might be objected that truth‐aptness tasks do not measure conceptual intuitions about morality in the sense specified above, i.e., pretheoretical dispositions to apply or refrain from applying concepts. This objection holds when it comes to moral concepts, such as right, wrong, good, bad, etc. However, it does not hold for the concept that is actually at issue in these tasks, namely the concept of a moral statement (or judgment). Consider the study by Pölzler and Wright (forthcoming‐a) that will be mentioned below. In this study subjects were asked whether they consider certain statements to be truth‐apt (e.g., “true”/“false” or “an opinion or attitude“), and whether they consider these statements to be moral (as opposed to, e.g., statements about science, social conventions, or personal preferences; see also Wright, 2018; Wright, Grandjean, & McWhite, 2013; Wright, McWhite, & Grandjean, 2014). If a subject takes all or most of the statements that she classified as moral to be truth‐apt then this suggests that the subject is disposed to apply the concept of a moral statement only to statements that are truth‐apt. In other words, the subject seems to have an intuition in favor of moral cognitivism.

11 Note that, according to some philosophers (e.g., Machery, 2017), the very notion of conceptual competency is philosophically inadequate.
